# Facile Preparation of Mesoporous MCM-48 Containing Silver Nanoparticles with Fly Ash as Raw Materials for CO Catalytic Oxidation

**DOI:** 10.3390/mi12070841

**Published:** 2021-07-19

**Authors:** Dong Tian, Yonghong Chen, Xiaoyong Lu, Yihan Ling, Bin Lin

**Affiliations:** 1 Huainan Engineering Research Center for Fuel Cells, Anhui Province Key Laboratory of Low Temperature Co-Fired Materials, Huainan Normal University, Huainan 232038, China; tiandong111@163.com (D.T.); chenyh@hnnu.edu.cn (Y.C.); xyonglu@163.com (X.L.); lyhyy@cumt.edu.cn (Y.L.); 2 School of Materials Science and Physics, China University of Mining and Technology, Xuzhou 221116, China; 3 School of Mechanical and Electrical Engineering, University of Electronic Science and Technology of China, Chengdu 611731, China

**Keywords:** fly ash, MCM-48, silver nanoparticles, composite, CO oxidation

## Abstract

An environmentally friendly method was proposed to prepare mesoporous Mobil Composition of Matter No.48 (MCM-48) using fly ash as the silica source. Silver nanoparticles were infiltrated on MCM-48 facilely by an in situ post-reduction method and evaluated as an effective catalyst for CO oxidation. The as-prepared MCM-48 and Ag/MCM-48 nanoparticles were characterized by XRD, N_2_ adsorption/desorption, and TEM. Investigations by means of XPS for Ag/MCM-48 were performed in order to illuminate the surface composition of the samples. Studies revealed the strong influence of the loading of Ag nanoparticles on catalysts in the oxidation of CO. CO conversion values for Ag/MCM-48 of 10% and 100% were achieved at temperatures of 220 °C and 270 °C, respectively, indicating that the Ag-decorated MCM-48 catalyst is extremely active for CO oxidation.

## 1. Introduction

Fly ash is a waste product from coal combustion in power plants. The large amount of SiO_2_ (50–70%) and Al_2_O_3_ (20–30%) in fly ash makes it suitable for preparing many industrial products. To improve the added value of fly ash, fly ash was used for the extraction of alumina and as a lightweight aggregate, zeolite, and adsorbent [1]. In addition, because the content of SiO_2_ is above 50%, using fly ash as a silica source to prepare mesoporous silica is an environmentally friendly approach to reuse the waste resources and has been regarded as a suitable skeleton to decorate nanostructured metals due to its large pores (2–50 nm) with a narrow distribution (the ease of macroscopic morphological control) and a high surface area. For example, Panek synthesized Mobil Composition of Matter No.41 (MCM-41) by a hydrothermal method under a long reaction time of 72 h using pristine fly ash [2]. Li successfully prepared MCM-41 under a high temperature (373 K) over 48 h [3]. It could be seen that MCM-41 could be successfully prepared from fly ash, but the long reaction time was not environmentally friendly and limited its widespread application.

Nanosized metal particles as quantum dots have aroused many scientists’ interest in recent years due to their unique properties attributed to quantum confinement or surface effects, which underlie its future applications in catalysis as well as in miniaturized electronic and optical devices [4]. CO oxidation is of practical importance for controlling the CO poisoning that can come from incomplete combustion processes, e.g., cigarette combustion. A CO oxidation catalyst has to be added to complete the combustion to remove the poisoning. The outstanding catalytic activities of Pt, Rh, and Au are widely recognized [5,6]. A high activity for CO oxidation at higher reaction temperatures (150–250 °C) can be obtained on Pt catalysts; however, the competitive adsorption of CO and O_2_ decreases the low-temperature activity of the catalysts [7]. Compared to the traditional catalysts, silver is gaining increasing attention. For instance, Huang et al. [8] used AgNO_3_ solution impregnation in the channels of SBA-15 followed by thermal decomposition to prepare Ag nanowires within SBA-15. Sajkowski and Boudart [9] have discussed the effects of silver particle size and surface orientation. Yin et al. [10] synthesized mesoporous Ag/MCM-41 and it displayed outstanding methyl glycolate and ethylene glycol selectivity. Gac et al. [4] prepared a silver-doped mesoporous silica MCM-41 material by the direct hydrothermal (DHT) and template ion exchange (TIE) methods. Bao et al. [11] found out that monodispersed silver nanoparticles with tunable sizes could be easily fabricated on silica-based materials by the in situ auto reduction route. Plyuto et al. [12] have reported Ag nanoparticles synthesized in template-structure mesoporous silica films. 

Although silver nanoparticle catalysts have found broad industrial and environmental applications, including the production of formaldehyde, epoxidation of ethylene, NO_x_ abatement, and deep hydrocarbon oxidation, the CO catalytic oxidation by silver nanoparticles is less often reported owing to the low catalytic activity [13,14]. It has been generally accepted that the use of reactive support can increase the activity of the silver catalyst by the oxygen spillover from the second compound or reactive support onto the silver catalyst [6]. Herein, an environmentally friendly approach was proposed by using fly ash as the silica source to prepare Mobil Composition of Matter No.48 (MCM-48) mesoporous molecular sieves. Hexamethylenetetramine (HMT) was used as an effective and mild reducing reagent, by which MCM-48-supported silver nanoparticles were easily obtained via impregnation and then reduction at a low temperature. The catalytic activities of CO oxidation for the prepared catalysts were systematically investigated. This study aimed to develop a new reduction method for synthesizing supported metal catalysts under mild conditions to overcome the traditional time-consuming synthesis process.

## 2. Experimental

### 2.1. Materials

The coal fly ash used in this study was obtained from the Luohe Power Plant, Huainan, China, and was used as obtained; its chemical composition was analyzed and is listed in Table 1. Apart from the main constituents, such as silica and alumina, the other impurities found in the ash were Fe_2_O_3_, CaO, K_2_O, MgO, and Na_2_O with contents of 3.7, 2.7, 1.5, 0.6, and 0.5%, respectively.

#### 2.1.1. Synthesis of Silica Resource

The coal fly ash was sieved to obtain a fine power with particles with a mesh size less than 100 um. It was then mixed well with NaOH powder at a 1:1.3 weight ratio and fused at 550 °C for 2 h. After cooling to room temperature, the obtained fused fly ash powder was mixed with water in a weight ratio of 1:4 and stirred for 24 h. The resulting suspension was then centrifuged and filtered to separate the suspended particles, and the supernatant was used for the synthesis of mesoporous silica.

#### 2.1.2. Synthesis of Mesoporous MCM-48 Support

MCM-48 support was synthesized following the procedure reported by sol-gel method. In a typical MCM-48 synthesis procedure, 0.749 g of cetyltrimethylammonium bromide (CTAB) was dissolved in 30 g of deionized water and stirred at 30 °C until all CTAB was dissolved. To the resulting solution, 15 mL ethanol was added, and the mixture was continuously stirred for 5 min. After the addition of 4 mL of 30 wt.% aq. NH_3_ H_2_O, which was after standing for 20 min, 15 mL of coal fly ash supernatant was added, and the mixture was stirred at 200–400 rpm for a further 2 h. The solution pH was adjusted with dilute acetic acid until the precipitate was obtained. The resulting white precipitate was filtered, washed with deionized water, and then collected and dried at 70–80 °C overnight. After drying, the mesoporous materials were calcined at 550 °C for 6 h with a heating rate of 2 °C/min to remove the surfactant.

#### 2.1.3. Preparation of Ag/MCM-48 by Post-Reduction Method

MCM-48-supported silver nanoparticles were obtained by adding 40 mL of 8.5 mM AgNO_3_ absolute alcohol solution to 0.35 g of MCM-48 support. The mixture was continuously stirred for 2 h at 30 °C. Reduction of Ag(I) was effectively performed by adding 0.216 g hexamethylenetetramine (HMT) to the above system, and then the mixture was stirred at 30 °C for 24 h in the dark. After filtering, washing with deionized water, and drying under vacuum at 50 °C for 10 h, the Ag/MCM-48 mesoporous silica was obtained.

### 2.2. Catalytic Activity 

The catalytic activity of the mesoporous silica-supported nano silver catalysts in CO oxidation was evaluated on a small fixed-bed microreactor operating under atmospheric pressure and an online GC using 50 mg of sample. The flow rate of the feed gas was 30 mL/min. The analysis of the effluent gas was conducted with an online FuLi9790 model gas chromatograph equipped with a Molecular Sieve 3 Å column and a thermal conductivity detector (TCD). The catalysts were directly exposed to reaction gas containing 2.5% (*v*/*v*) CO, 10% (*v*/*v*) O_2_, and 87.5% (*v*/*v*) N_2_. The conversion of CO was calculated from the change in CO concentration in the inlet and outlet gases.

## 3. Results and Discussion

### 3.1. X-Ray Diffraction Analysis

**Figure 1:**
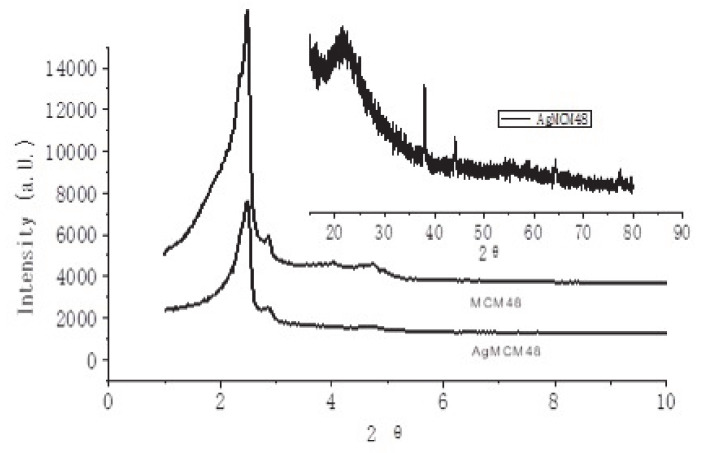
Powder XRD patterns of MCM-48 and Ag/MCM-48. The inset diagrams show the high-angle PXRD of Ag/MCM-48.

The small-angle X-Ray Diffraction (XRD) patterns of mesoporous MCM-48 and Ag/MCM-48 are shown in Figure 1. It can be seen that there were no substantial changes after the loading with Ag. From the high-angle XRD patterns, silver-loading samples show XRD reflections typical of metallic silver, with four intense diffraction peaks corresponding to the (111), (200), (220), and (311) lattice planes of the cubic structure of Ag. It also can be seen that Ag/MCM-48 prepared by the post-reduction technique presented weaker diffraction peaks in the high-angle XRD patterns (such as (111) peak of Ag), which shows that MCM-48-supported Ag nanoparticles presented a smaller size.

### 3.2. Transmission Electron Micrographs Characterization

**Figure 2:**
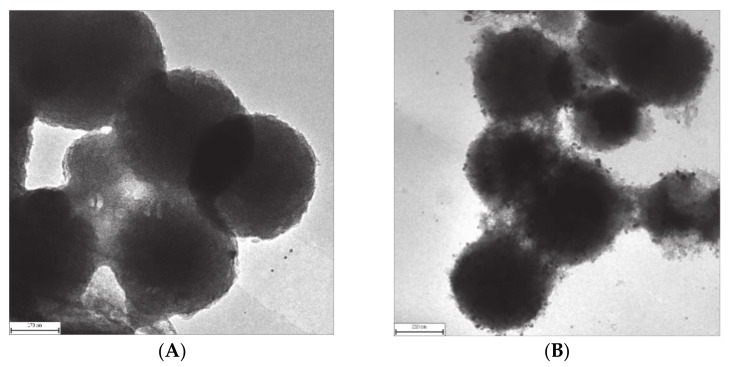
TEM images of various samples (**A**): MCM-48; (**B**): Ag/MCM-48.

Transmission Electron Micrographs (TEM) images of the final calcined samples are shown in Figure 2. Transmission electron micrographs (TEM) give a visualization of the particle morphology. It is clear from Figure 2A that the mesoporous silica existed as average silica spheres. The pore structure was regular over the whole particle. Figure 2B shows the very uniform dispersion of Ag particles within the MCM-48 spheres. It also was clear from TEM that some of the nanoparticles led to localized swelling of the mesopore wall structure, probably because the local concentration of the precursor exceeded the optimum for restricted growth within the channel. The nanoparticles had an average size of 10 nm, as can be seen from the images. This shows that the Ag precursor could diffuse well into the micelle structure, aided by the HMT at 35 °C.

### 3.3. N_2_ Adsorption/Desorption Isotherms and Pore Size Distributions

**Figure 3:**
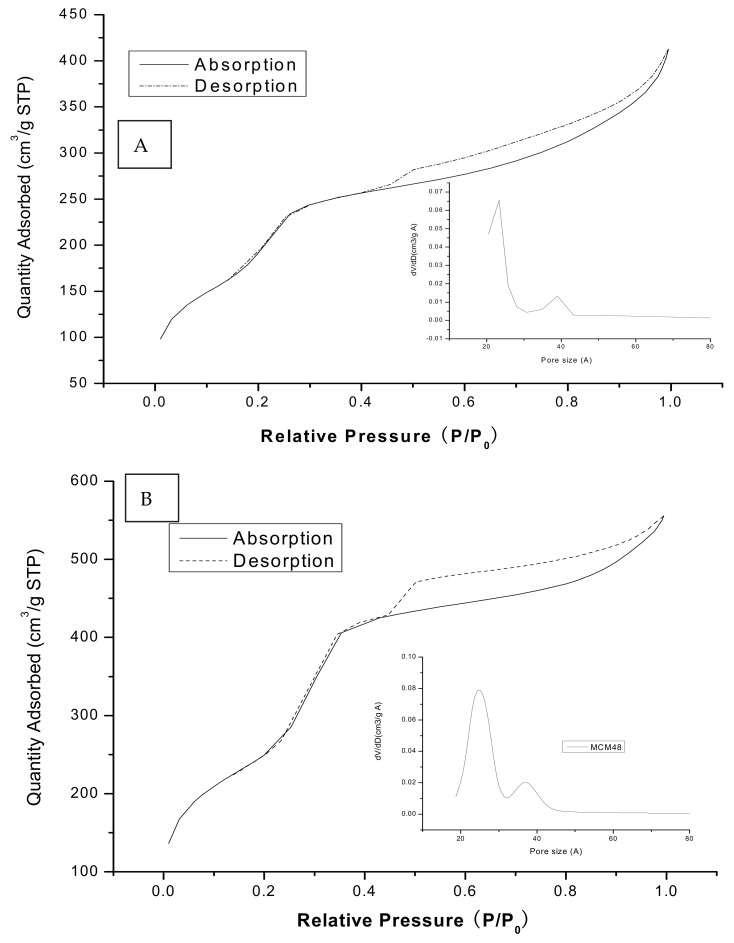
The adsorption/desorption isotherms for Ag/MCM-48 (**A**) and MCM-48 (**B**).

Both Ag/MCM-48 and MCM-48 exhibited similar N_2_-sorption isotherms of type IV, as shown in Figure 3A,B. They exhibited a distinct step over a narrow range of relative pressures P/P0 = 0.4–0.52, which is typical for ordered mesoporous silica materials. The pure silica MCM-48 exhibited a large surface area SBET = 1060 m^2^/g and primary pore volume V_p_ = 0.95 cm^3^/g. The total surface area decreased to 860 m^2^/g for Ag/MCM-48, and the primary mesopore volume (V_p_) decreased to 0.72 cm^3^/g. These observations imply that part of the pore space was loaded and blocked with Ag nanoparticles. The BJH pore size distribution plots are shown in the insets of Figure 3A,B. The very narrow distribution for the pure silica MCM-48 indicates the uniformity of mesopores. The silver-loaded material was characterized by a somewhat broader distribution. Interestingly, both Ag/MCM-48 and MCM-48 exhibited a bimodal pore size distribution composed of pores with small (2.3 nm) and large (3.9 nm) diameters for Ag/MCM-48 and small (2.5 nm) and large (3.6 nm) diameters for MCM-48, respectively.

### 3.4. Catalytic Oxidation of CO 

**Figure 4:**
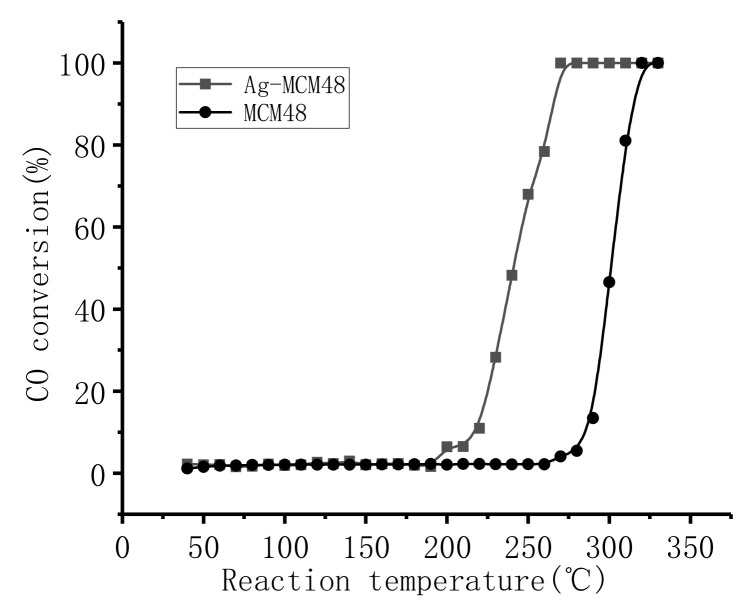
CO oxidation of MCM-48 and Ag/MCM-48.

These samples exhibited different catalytic behavior in CO oxidation, as shown in Figure 4, which shows the conversion of CO over the catalysts as a function of the reaction temperature. Conversion of CO for the Ag/MCM-48 sample remained at a very low level over the temperature range of 40–150 °C, implying that Ag nanoparticles are not active for catalytic oxidation CO at a relatively low-temperature host in MCM-48 materials, although several studies have indicated that CO can be oxidized over silver even below room temperature. Ag/MCM-48 becomes active at a temperature of around 200 °C, with a CO conversion of 6%, and then achieves 100% CO conversion at 270 °C.

However, the pure silica MCM-48 showed lower catalytic activity, becoming active at a higher temperature (270 °C), with a CO conversion of 5%; it then also achieved 100% CO conversion at 320 °C. This implies that finer Ag nanoparticles loaded on mesoporous silica exhibit better catalytic performance for CO oxidation.

We proposed that the observed Ag nanoparticle size–reaction temperature relation in the CO oxidation catalyzed by Ag/mesoporous silica catalysts might be attributed to the fine and non-aggregated Ag nanoparticles that are more chemically active. However, at high reaction temperatures, oxygen is easily thermally activated and no longer depends on the Ag nanoparticle size; as a result, CO conversion reaches 100%. Further investigations are required for the understanding of the observed Ag particle size–reaction temperature relation in the CO oxidation catalyzed by Ag/mesoporous silica catalysts, and studies have revealed a strong influence of the pretreatment and reduction conditions on the performance of these Ag/mesoporous silica catalysts in the oxidation of CO; these studies are ongoing in our laboratory.

## 4. Conclusions

Mesoporous MCM-48 was synthesized with the supernatant solution as silicate species by an alkali fusion method for extraction from fly ash in a power plant. Silver nanoparticles were loaded on MCM-48 facilely prepared by the impregnation and in situ post-reduction method. The as-prepared MCM-48 and Ag/MCM-48 nanoparticles were characterized by XRD, N_2_ adsorption/desorption, and TEM. Investigations by means of XPS for Ag/MCM-48 were performed in ordered to illuminate the surface composition of the samples. In addition, the catalytic activities towards CO oxidation were investigated. Studies revealed the strong influence of the loading of Ag nanoparticles on catalysts in the oxidation of CO. The CO conversion values for Ag/MCM-48 of 10% and 100% were achieved at temperatures of 220 °C and 270 °C, respectively.

## Figures and Tables

**Table 1:** Elemental compositions (wt.%) of coal fly ash, fused fly ash powder, and the materials synthesized.

SiO_2_	Al_2_O_3_	Fe_2_O_3_	CaO	K_2_O	MgO	Na_2_O
62%	25%	3.7%	2.7%	1.5%	0.6%	0.5%
